# 2376 Efficacy of a 4-part program on brain development

**DOI:** 10.1017/cts.2018.251

**Published:** 2018-11-21

**Authors:** Emily Silver, Nancy Michael

**Affiliations:** Indiana University School of Medicine

## Abstract

OBJECTIVES/SPECIFIC AIMS: (1) Provide basic brain knowledge about development and resiliency. (2) Develop an understanding of how a mother can impact a child’s brain development. (3) Foster a sense of agency to increase the likelihood that a mother will enact positive changes. (4) Develop the ability to recognize a connection between one’s own behaviors and a child’s development and behaviors. METHODS/STUDY POPULATION: Tested the efficacy of a 4-week intervention program on neurodevelopment for homeless mothers. Mothers (n=4) residing at the Center for the Homeless in South Bend, IN were recruited. Used community partner feedback, weekly surveys, and pre/post tests to look at changes in basic content knowledge, behavioral change, and self-efficacy. RESULTS/ANTICIPATED RESULTS: Preliminary results indicate an increase in knowledge about neurodevelopment, although results on behavioral changes are inconclusive. The program is anticipated to run a second time with a new group of parents residing in the Center for the Homeless to increase sample size. DISCUSSION/SIGNIFICANCE OF IMPACT: Anticipated that the results will add to the existing literature concerning effective interventions in strengthening parenting and neuroscience knowledge in vulnerable populations.

